# Exosome and biotherapeutic strategies for dermatological and oncological skin complications

**DOI:** 10.1080/07853890.2026.2673184

**Published:** 2026-05-15

**Authors:** Hani Tauseef, Wang Yun-jie, Abubakar Shahid, Ayesha Siddiqa, Ci Chao, Yuan Tao

**Affiliations:** ^a^ Department of Dermatology, Yijishan Hospital, the First Affiliated Hospital of Wannan Medical College, Wuhu, Anhui Province, China; ^b^ Xi ‘an Jiaotong University, Xian, Shanxi Province, China; ^c^ Wannan Medical College, Wuhu, Anhui Province, China; ^d^ Ministry of Education, Key Laboratory of Dermatology (Anhui Medical University), Hefei, Anhui, China

**Keywords:** Exosome, skin disease, inflammation, therapy

## Abstract

**Background:**

Exosomes are nanosized extracellular vesicles (30–150 nm) that mediate intercellular communication by transferring bioactive molecules, including proteins, lipids, and nucleic acids. Their ability to regulate inflammation, immune responses, angiogenesis, and tissue regeneration has positioned them as promising candidates for the management of dermatological diseases and oncologic skin complications.

**Methods:**

A comprehensive literature review was conducted using PubMed and Web of Science databases (2020–2025). Relevant preclinical studies, clinical trials, and mechanistic investigations evaluating exosome biology, therapeutic applications, and biotherapeutic strategies in dermatological and oncologic skin conditions were included and analysed.

**Results:**

Exosome-based therapies, particularly those derived from mesenchymal and adipose-derived stem cells, demonstrate significant regenerative and immunomodulatory effects. These include enhanced wound healing, reduced inflammation, improved extracellular matrix remodelling, and modulation of immune pathways. Therapeutic benefits have been reported in chronic wounds, inflammatory dermatoses, pigmentary disorders, skin ageing, acne scarring, and treatment-related skin toxicities. In oncologic contexts, exosomes exhibit dual roles, contributing to both tissue repair and tumour progression depending on their origin. Despite strong preclinical evidence, clinical translation remains limited due to challenges in standardisation, scalability, and safety evaluation.

**Conclusions:**

Exosome-based biotherapeutics represent a promising, cell-free strategy for managing complex dermatological and oncologic skin conditions. Future progress will depend on standardised manufacturing protocols, rigorous clinical trials, and long-term safety validation to enable effective clinical application.

## 1. Introduction

In this context, exosomes, nanosized extracellular vesicles (≈ 30–150 nm) secreted by virtually all cell types, have emerged as promising mediators of tissue repair and immune modulation. These vesicles carry a complex cargo of proteins, lipids, RNAs, and signaling molecules reflecting the state of their parent cells; through paracrine interactions they can modulate inflammation, promote angiogenesis, stimulate proliferation and migration of skin cells (keratinocytes, fibroblasts), and regulate ECM remodeling [1–3]. Exosomes are common membrane-bound nanovesicles that carry various bioactive substances, which are involved in intercellular communication and cellular signal transduction and changes in cell or tissue metabolism [4]. Because of these multifaceted effects, exosomes represent a compelling cell-free therapeutic modality, potentially avoiding risks of cell-based therapies (e.g. immunogenicity, tumorigenicity) while delivering the regenerative signals of cell therapy [1,5].

Preclinical and translational research increasingly supports exosome-based interventions in dermatology. For instance, reports have demonstrated accelerated wound closure, improved vascularization, enhanced re-epithelialization, reduced inflammation, and better ECM restoration following administration of exosomes derived from mesenchymal stem cells (MSCs) or adipose-derived stem cells (ADSCs) [1,6,7], Recently, exosomes have also shown efficacy in preventing or reducing skin injury linked to radiation therapy: a study demonstrated that ADSC-derived exosomes upregulate hyaluronic acid synthase 1 (HAS1), activate TGF-β/Smad2/3 signaling, and thus promote repair of radiation-induced dermatitis in a rodent model [7]. These findings underscore the potential of exosome-based biotherapeutics to address both non-malignant dermatologic conditions and oncologic skin toxicities.

Moreover, exosome-based strategies are part of a broader shift toward biotherapeutics including growth-factor treatments, cytokine modulators, peptide/nanovesicle delivery systems that aim to restore skin homeostasis through biologically rational mechanisms. Reviews suggest that such interventions may complement or outperform traditional therapies, especially in complex skin pathologies characterised by chronic inflammation, impaired healing, or immune-mediated damage [2,3,5]. Compared with whole-cell therapy, exosomes offer advantages in storage, reproducibility, safety, and regulatory feasibility.

Nonetheless, despite promising preclinical data, notable challenges remain. There is considerable heterogeneity in exosome isolation, purification and characterisation methods; lack of standardised protocols hinders reproducibility and clinical translation [3]. In addition, crucial questions about optimal dosing, delivery routes, biodistribution, long-term safety, and the risk of unwanted effects (e.g. pro-fibrotic or pro-tumorigenic responses) remain largely unresolved [5].

Given the high unmet need in dermatological and oncologic skin complications, the biological plausibility of exosome-mediated repair and immune regulation, and increasing preclinical evidence, biotherapeutic strategies centred on exosomes represent a highly promising direction. This review aims to synthesise current evidence, delineate mechanisms of action, assess therapeutic potential and limitations, and outline future directions to facilitate translation into clinical practice. Exosomes in dermatological research can be broadly categorised into endogenous (self-produced) and exogenously administered (therapeutic) vesicles, a distinction that is critical for accurate interpretation of their biological roles. Endogenous exosomes are secreted by resident skin cells or tumour cells and often reflect pathological states, contributing to inflammation, immune dysregulation, or tumour progression. In contrast, exogenously applied exosomes, typically derived from mesenchymal stem cells (MSCs), adipose-derived stem cells (ADSCs), or other controlled sources, are enriched in regenerative and immunomodulatory cargo and are utilised for therapeutic purposes. Throughout this review, this distinction is maintained to clearly differentiate between disease-driving and therapeutically beneficial exosomal functions.

## 2. Biology of exosomes

**Figure 1.**
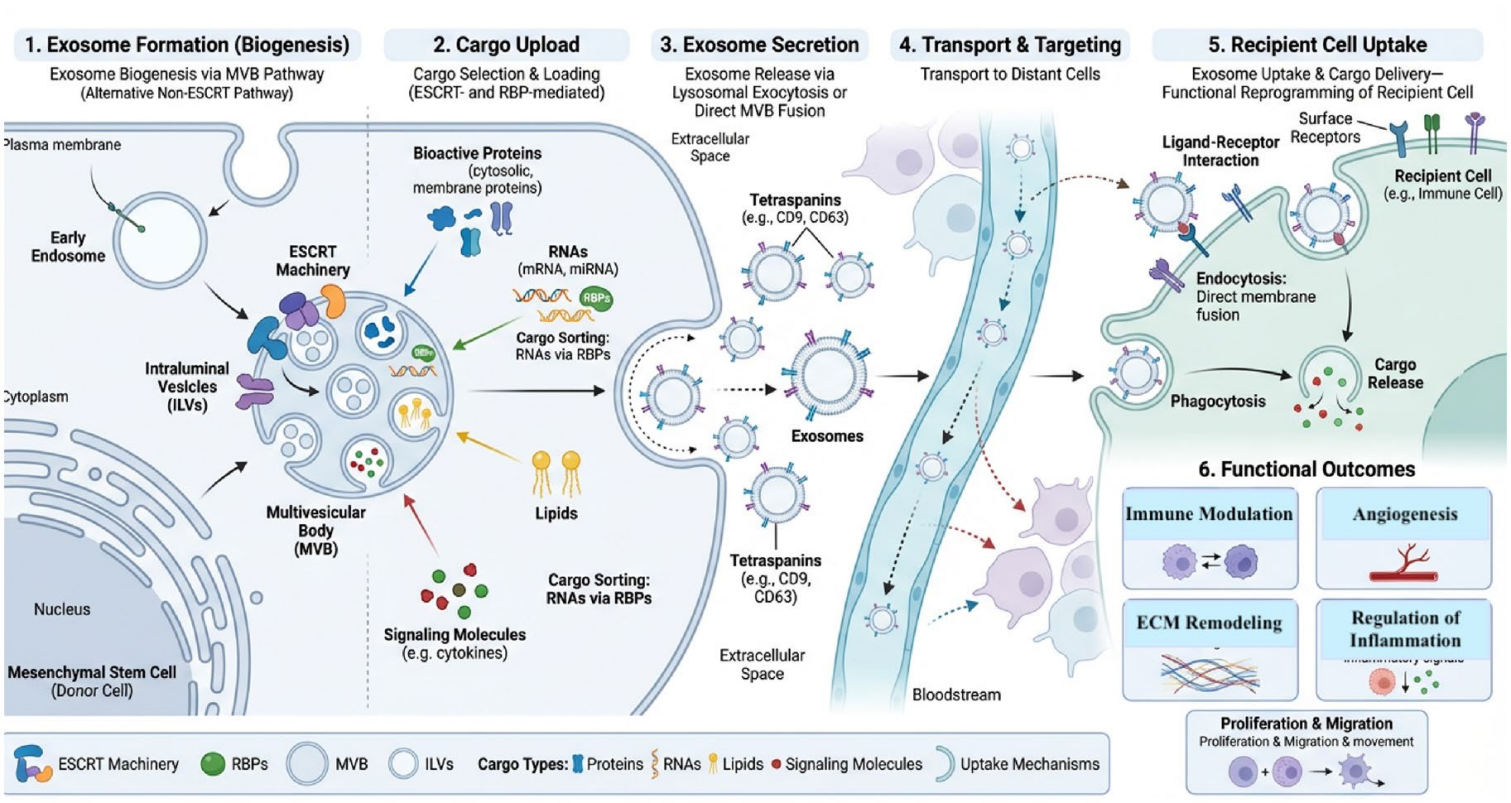
Biology of Exosome formation, cargo loading, and delivery to recipient cells.

Exosomes are small membranous extracellular vesicles, typically ranging from approximately 40 to 160 nm in diameter, that are widely distributed in biological fluids including blood, saliva, tears, and urine, and are secreted by a variety of cell types [8]. They function as important mediators of intercellular communication by transferring bioactive cargo between cells. Exosome formation is a highly regulated, multistep process involving double invagination of the plasma membrane and the generation of intracellular multivesicular bodies (MVBs) containing intraluminal vesicles (ILVs). Initially, the plasma membrane undergoes inward budding to form early-sorting endosomes (ESEs), which incorporate cell surface proteins and extracellular components. These early endosomes can also receive contributions from intracellular compartments such as the endoplasmic reticulum and trans-Golgi network, further influencing their molecular composition [8]. ESEs subsequently mature into late-sorting endosomes (LSEs) and ultimately into MVBs. During this maturation process, the limiting membrane of the endosome undergoes inward budding, resulting in the formation of ILVs within the lumen of MVBs. These ILVs represent the precursors of exosomes and are later released into the extracellular environment upon fusion of MVBs with the plasma membrane [8]. The formation of ILVs is mediated by both ESCRT-dependent and ESCRT-independent pathways. The ESCRT (endosomal sorting complex required for transport) machinery, comprising ESCRT-0, -I, -II, and -III complexes along with VPS4, plays a key role in cargo recognition, membrane budding, and vesicle scission. In this process, upstream ESCRT components (ESCRT-0 to -II) are involved in cargo sequestration at the endosomal membrane, while downstream components (ESCRT-III and VPS4) facilitate membrane remodeling and vesicle formation [9]. In addition to its structural role, ILV formation contributes to the regulation of cellular signaling by facilitating the sequestration and degradation of membrane receptors, thereby attenuating growth factor-mediated signaling pathways [9]. However, accumulating evidence suggests that ESCRT machinery is not strictly required for exosome biogenesis. Studies have demonstrated that disruption of key ESCRT components does not completely abolish exosome secretion, indicating the presence of ESCRT-independent mechanisms [10]. These alternative pathways include ceramide-dependent lipid microdomain formation, in which sphingomyelin hydrolysis by sphingomyelinase generates ceramide, promoting membrane curvature and ILV budding, as well as tetraspanin-mediated sorting mechanisms that facilitate cargo clustering and vesicle formation [10]. Following ILV formation, MVBs undergo a critical fate decision. They may either fuse with lysosomes or autophagosomes for degradation of their cargo or traffic toward the plasma membrane for exosome release [11]. Fusion of MVBs with the plasma membrane results in the exocytosis of ILVs into the extracellular space, at which point they are termed exosomes [12]. Lysosomes represent a major destination for MVBs, where enclosed ILVs can be degraded. However, under certain conditions, lysosomal exocytosis may also contribute to extracellular vesicle release, further linking intracellular degradation pathways with extracellular communication [13]. Exosomes act as cargo delivery vehicles, transporting a diverse array of biomolecules, including proteins, lipids, DNA fragments, mRNA, and microRNAs, which reflect the physiological or pathological state of the parent cell. Through transfer of this cargo, exosomes regulate gene expression and signaling pathways in recipient cells. They mediate both autocrine and paracrine intercellular communication, which is essential for maintaining tissue homeostasis and coordinating cellular responses. These processes are closely linked with intracellular trafficking pathways, including the ER-Golgi secretory pathway and secretory autophagy, which facilitate the release of signaling molecules such as cytokines and growth factors into the extracellular environment [14] The overall process of exosome biogenesis, cargo loading, secretion and delivery to reciepient cells is illustrated in Figure 1.

### 2.1. Cargo types

Exosomes encapsulate a heterogeneous molecular cargo that reflects the physiological and pathological state of their parent cell, including proteins (membrane and cytosolic proteins, enzymes, and signaling receptors), multiple RNA species (mRNA, microRNA, and other small RNAs), DNA fragments, lipids, and metabolites [15]. More specifically, exosomal cargo comprises transmembrane proteins such as tetraspanins (CD9, CD63, CD81), cytosolic proteins, enzymes, signaling receptors, lipids, metabolites, and diverse nucleic acids, including mRNA, microRNA (miRNA), long non-coding RNA (lncRNA), circular RNA (circRNA), and both genomic and mitochondrial DNA fragments [16]. This complex molecular composition enables exosomes to function as biologically active carriers capable of modulating gene expression, metabolic pathways, and intracellular signalling networks in recipient cells. Importantly, exosomal cargo loading is not a passive or random process. Increasing evidence demonstrates that RNA and protein incorporation into exosomes is highly selective and regulated by dedicated molecular sorting mechanisms, including RNA-binding proteins, sequence-specific motifs, and lipid-driven pathways [15]. Proteomic and transcriptomic analyses further support this concept by showing enrichment of specific proteins and RNA species within exosomes compared to their parent cells, indicating the presence of active and selective cargo-sorting machinery [17]. Through this regulated cargo packaging, exosomes can effectively reprogram recipient cells, influencing cellular behaviour, immune responses, and tissue homeostasis.

#### 2.1.1. RNA cargo and sequence-dependent sorting

RNA is among the most functionally significant components of exosomes. Exosomal RNAs are protected from extracellular RNases by the vesicular lipid bilayer, allowing stable transport through biological fluids and enabling intercellular transfer of genetic information. Specific RNA species are selectively enriched within exosomes through sequence-dependent mechanisms involving RNA motifs (often termed EXOmotifs) that are recognised by RNA-binding protein (Huang Z). RNA-binding proteins such as heterogeneous nuclear ribonucleoproteins (hnRNPA2B1), Y-box binding protein-1 (YBX1), Argonaute-2, and major vault protein have been shown to bind defined RNA sequence motifs and mediate their sorting into intraluminal vesicles during multivesicular body formation. These complexes guide the trafficking of selected miRNAs and mRNAs to the endosomal membrane, where they are incorporated into budding vesicles [18].

#### 2.1.2. Lipid- and ceramide-dependent cargo loading

In addition to protein-mediated mechanisms, lipid-driven processes play an important role in cargo selection. Neutral sphingomyelinase-2 (nSMase2) regulates ceramide production, which promotes membrane curvature and intraluminal vesicle formation while simultaneously influencing the sorting of specific miRNAs into exosomes. Alterations in sphingolipid metabolism have been shown to change both exosome quantity and RNA composition, highlighting the integration between membrane lipid dynamics and molecular cargo selection [19].

#### 2.1.3. Protein cargo and membrane microdomain sorting

Protein sorting into exosomes is also a selective process mediated by endosomal microdomains enriched in tetraspanins, ESCRT components, and lipid rafts. Ubiquitinated cytosolic proteins are commonly recognised by ESCRT machinery for inclusion into intraluminal vesicles, while tetraspanin-enriched microdomains serve as platforms for clustering specific membrane proteins and receptors destined for exosomal secretion. These mechanisms ensure that exosomes carry signalling-competent protein complexes capable of interacting with recipient cells [17].

#### 2.1.4. Functional implications of cargo heterogeneity

The selective packaging of nucleic acids, proteins, and lipids enables exosomes to act as vectors of horizontal molecular transfer between cells. Once internalised by recipient cells through endocytosis, phagocytosis, or membrane fusion, exosomal cargo can reprogram cellular phenotypes by modulating transcriptional networks, immune responses, angiogenesis, and tumour microenvironment signalling. This ability to deliver functional biomolecules across tissue barriers underlies both the physiological role of exosomes in homeostasis and their pathological contribution to cancer progression, inflammatory diseases, and dermatological disorders [16].

### 2.2. Mechanisms of action

Exosomes mediate intercellular communication primarily through paracrine signalling, enabling the transfer of biological signals between adjacent and distant cells. They exert their effects through three principal mechanisms: receptor-ligand interactions at the cell surface, endocytic internalisation, and direct membrane fusion with recipient cells, thereby delivering functional cargo such as miRNAs, mRNAs, and proteins that reprogram cellular behaviour [20,21]. Exosomes exert their biological effect through receptor-mediated signalling, endocytosis, and direct membrane fusion as summarised in Figure 1.

#### 2.2.1. Surface receptor engagement and signalling activation

Exosomes can interact with recipient cells by binding to membrane receptors through ligand-receptor recognition, thereby triggering intracellular signalling cascades without requiring vesicle internalisation. This mechanism allows rapid modulation of key pathways such as MAPK, PI3K-Akt, and NF-κB signalling, influencing cellular proliferation, survival, and inflammatory responses.

Through these receptor-mediated interactions, exosomes can function analogously to soluble cytokines while maintaining the spatial organisation and multivalent presentation of signalling molecules on their membrane surface [21]**.**

#### 2.2.2. Endocytosis and intracellular trafficking

In many cell types, exosomes are internalised through energy-dependent endocytic pathways, including clathrin-mediated endocytosis, caveolin-dependent uptake, macropinocytosis, and phagocytosis. Following uptake, exosomes are trafficked to early endosomes, where they may either fuse with endosomal membranes to release their bioactive cargo into the cytosol or be directed toward lysosomal degradation.

The intracellular trafficking route determines the efficiency, localisation, and duration of exosome-mediated signalling. Notably, exosomes can facilitate distal intracellular transport of encapsulated cargo and, in some contexts, enable passage across biological barriers such as the blood-brain barrier [22,23]. Furthermore, exosome heterogeneity influences uptake and trafficking efficiency, with vesicles derived from different cell types exhibiting distinct delivery capabilities due to variations in surface protein composition and adhesion properties [22,23].

#### 2.2.3. Direct membrane fusion

In addition to endocytosis, exosomes may directly fuse with the plasma membrane of recipient cells, releasing their cargo into the cytoplasm. This mechanism provides an efficient route for rapid intracellular delivery of functional molecules, particularly in target cells with compatible membrane properties [21].

#### 2.2.4. Modulation of the extracellular microenvironment

Beyond direct cellular interactions, exosomes actively modulate the extracellular milieu. They interact with extracellular matrix (ECM) components, regulate matrix metalloproteinase activity, and facilitate the presentation and stabilisation of growth factors. Through these mechanisms, exosomes influence tissue remodelling, angiogenesis, and cellular migration - processes that are critical in wound healing, fibrosis, and tumour invasion [24].

#### 2.2.5. Functional consequences in tissue homeostasis and repair

Through the integration of receptor-mediated signalling, intracellular cargo delivery, and extracellular matrix modulation, exosomes regulate key biological processes, including immune responses (activation or suppression), angiogenesis, cellular proliferation, migration, and oxidative stress responses. These coordinated effects play essential roles in maintaining tissue homeostasis and orchestrating regenerative processes following injury or therapeutic intervention [20,25].

#### 2.2.6. Delivery of exosomes to distant cells

In addition to local (paracrine) signalling, exosomes possess the capacity to mediate long-distance intercellular communication. They can enter systemic circulation *via* blood and lymphatic vessels and subsequently home to distant tissues in a regulated manner. Their biodistribution is governed by surface molecules such as integrins, tetraspanins, and adhesion receptors, which confer tissue tropism and enable selective targeting of recipient cells.

Following systemic transport, exosomes interact with endothelial barriers and are internalised by distant cells through receptor-mediated binding or endocytosis, facilitating the transfer of bioactive signals across organ systems. This targeted delivery mechanism is particularly relevant in pathological conditions, where exosomes contribute to processes such as metastatic niche formation, immune modulation, and tissue repair at sites remote from their origin [26]. Their systemic transport and targeted delivery to distant tissues are depicted schematically in Figure 1.

### 2.3. Source-specific differences

#### 2.3.1. Stem-cell (MSC/epidermal)-derived exosomes

Exosomes from mesenchymal or epidermal stem cells are enriched in regenerative miRNAs, anti-inflammatory mediators, and pro-angiogenic proteins. Preclinical studies show MSC-exosomes promote fibroblast proliferation, keratinocyte migration, collagen deposition, angiogenesis, and reduced inflammation, effects that underpin their interest for wound healing and skin regeneration while offering a cell-free safety advantage over whole-cell therapies [27–29].

#### 2.3.2. Fibroblast-derived exosomes

Fibroblast exosomes carry ECM-modifying enzymes, growth factors, and metabolic regulators that directly influence dermal remodelling and angiogenesis. Several recent experimental reports indicate fibroblast-exosomes enhance re-epithelialization and neovascularisation in wound models and may be especially relevant for addressing matrix repair and scar modulation [30].

#### 2.3.3. Tumour-derived exosomes (TDEs)

Tumour-secreted exosomes are skewed toward oncogenic cargo (onco-proteins, immunomodulatory factors, pro-angiogenic miRNAs) and play complex roles in the tumour microenvironment: promoting angiogenesis, remodelling stroma, facilitating immune evasion, and preparing pre-metastatic niches. Importantly for translational dermatology, TDEs may both exacerbate tumour-associated skin pathology and carry useful biomarkers but also pose safety concerns if used therapeutically without rigorous characterisation [31–33]. Exosomes are versatile, source-dependent mediators whose cargo and surface composition determine therapeutic potential and safety. Standardised methods for isolation, rigorous molecular characterisation, and source-specific functional profiling are essential prerequisites for clinical translation of exosome-based biotherapeutics in dermatologic and oncologic skin applications [15].

## 3. Pathological role of endogenous exosomes in dermatological diseases

Endogenous exosomes are nanosized extracellular vesicles secreted by skin-resident cells, including keratinocytes, melanocytes, fibroblasts, and immune cells, and play essential roles in maintaining cutaneous homeostasis. Under pathological conditions, however, their cargo composition and signalling functions become altered, enabling them to contribute actively to disease progression [34,35]. In dermatological disorders, endogenous exosomes act as carriers of microRNAs, cytokines, and signalling molecules that mediate intercellular communication and propagate disease-related pathways. In inflammatory dermatoses such as psoriasis, keratinocyte-derived exosomes promote inflammatory signalling, including activation of NF-κB and MAPK pathways and increased cytokine production, thereby amplifying immune dysregulation [34]. In vitiligo, exosomal microRNAs play a key role in disease pathogenesis by regulating immune imbalance, oxidative stress, and melanocyte-keratinocyte interactions, ultimately contributing to melanocyte dysfunction and loss [36]. Additionally, dysregulated exosome signalling influences oxidative stress responses, apoptosis, and extracellular matrix remodelling, which are central mechanisms in both inflammatory and degenerative skin diseases [35,37]**.** Overall, endogenous exosomes function as active mediators of dermatological disease pathogenesis, highlighting the importance of clearly distinguishing them from exogenously administered therapeutic exosomes.

## 4. Role of exosomes in dermatological conditions

### 4.1. Wound healing

Wound healing is a complex, tightly regulated process essential for restoring epidermal barrier integrity following skin injury [38]. Mesenchymal stem cells (MSCs), particularly adipose-derived mesenchymal stem cells (ADSCs), play a significant role in tissue regeneration due to their accessibility and capacity to secrete bioactive factors involved in repair processes [39–43]. In this context, exosome-based therapies have emerged as promising cell-free approaches for enhancing wound healing outcomes.

### 4.2. Mechanisms of action

Exogenously administered exosomes, particularly those derived from MSCs and ADSCs, promote wound repair through coordinated modulation of inflammation, cellular migration, and extracellular matrix (ECM) remodelling. As outlined in earlier sections, exosomes deliver bioactive cargo, including miRNAs and proteins that reprogram recipient cells toward a regenerative phenotype [2]. Exosomes enhance keratinocyte and fibroblast migration and proliferation, thereby accelerating re-epithelialization and tissue repair. They also stimulate collagen deposition and regulate ECM remodelling, contributing to the restoration of dermal architecture. In addition, exosomes promote angiogenesis, which is essential for supplying nutrients and oxygen to healing tissues [41,42]. At the immune level, exosomes modulate inflammatory responses by reducing pro-inflammatory cytokines and promoting macrophage polarisation toward an anti-inflammatory (M2) phenotype, facilitating resolution of inflammation. Furthermore, MSC-derived exosomes support fibroblast activation and matrix production, including collagen and fibronectin synthesis, which are critical for structural repair following injury [44].

### 4.3. Preclinical evidence

Preclinical studies provide strong evidence supporting the role of exosomes in wound healing. MSC-derived exosomes have been shown to accelerate wound closure in animal models by enhancing keratinocyte and fibroblast activity, increasing collagen deposition, and promoting angiogenesis [41,42]. Additionally, exosome-based therapies have demonstrated the ability to modulate immune responses and improve tissue regeneration through delivery of regulatory miRNAs and proteins [2]. Emerging approaches using engineered delivery systems, such as hydrogels and ECM-based scaffolds, have further improved exosome retention and therapeutic efficacy *in vivo* [45]. Collectively, these findings indicate that exosomes can effectively coordinate multiple phases of wound healing, including inflammation resolution, tissue formation, and remodeling.

### 4.4. Clinical and translational considerations

Despite promising preclinical outcomes, clinical evidence remains limited. Current findings are largely derived from experimental models, and further clinical studies are required to establish efficacy, optimal dosing strategies, and delivery methods. Exosome-based therapies may offer advantages over cell-based approaches, including reduced immunogenicity and improved safety profiles. However, challenges such as variability in exosome source, scalability of production, and lack of standardised protocols remain significant barriers to clinical translation. While exosome-mediated effects may contribute to reduced scar formation and improved biomechanical properties of healed tissue, these outcomes require validation in well-designed clinical trials.

### 4.5. Skin ageing/photoaging

Skin ageing is a multifactorial process driven by intrinsic factors such as genetic and hormonal influences, as well as extrinsic factors including ultraviolet (UV) radiation and environmental exposure [46]. Clinically, it is characterised by thinning, dryness, reduced elasticity, dyspigmentation, and wrinkle formation. Photoaging, a major component of extrinsic ageing, results from cumulative UV-induced damage leading to oxidative stress, collagen degradation, elastosis, and increased susceptibility to skin malignancies [47,48]. Exosome-based therapies, particularly those derived from stem cells and dermal fibroblasts, have emerged as promising strategies for mitigating ageing-related changes in the skin.

#### 4.5.1. Mechanisms of action

Exosomes exert anti-ageing and anti-photoaging effects through modulation of oxidative stress, inflammation, and extracellular matrix homeostasis. They enhance collagen synthesis while suppressing matrix-degrading enzymes such as matrix metalloproteinases (MMPs), thereby preserving dermal structure and elasticity [49,50]. In UV-damaged skin, exosomes reduce reactive oxygen species (ROS) levels, attenuate inflammatory signalling, and decrease markers of DNA damage and cellular senescence. They also regulate key signalling pathways, including TGF-β/Smad and antioxidant pathways, which are essential for maintaining dermal integrity and promoting tissue repair [48,49]. Additionally, exosomes derived from dermal fibroblasts have been shown to maintain antioxidant enzyme activity and support ECM protein production, including collagen and elastin, thereby contributing to improved skin structure and function [50].

#### 4.5.2. Preclinical evidence

Preclinical studies demonstrate that exosome-based therapies can effectively counteract UV-induced skin damage. Exosomes derived from human dermal fibroblasts have been shown to reduce oxidative stress, prevent DNA damage, and inhibit cellular senescence in fibroblasts exposed to UVB radiation. In animal models, these effects are associated with preservation of dermal collagen and elastin, suppression of MMP-1 expression, and reduction in wrinkle formation and transepidermal water loss [50]. Emerging therapeutic strategies have further enhanced the efficacy of exosome delivery. For example, microneedle-based transdermal delivery systems combining MSC-derived exosomes with antioxidants have demonstrated improved skin regeneration following UV damage. In addition, plant-derived nanovesicles, such as those derived from grapes, have shown protective and reparative effects in preclinical photoaging models, suggesting potential scalable alternatives [49,51].

#### 4.5.3. Clinical and translational considerations

Although preclinical findings are encouraging, clinical evidence supporting the use of exosomes in skin ageing and photoaging remains limited. Most available data are derived from experimental and animal studies, and further clinical trials are required to validate therapeutic efficacy and long-term safety. Exosome-based therapies offer potential advantages, including cell-free delivery of regenerative signals and reduced risk of immune rejection. However, challenges such as heterogeneity of exosome preparations, lack of standardised manufacturing processes, and variability in delivery methods must be addressed before routine clinical application. Furthermore, while early findings suggest improvements in skin elasticity and wrinkle reduction, these outcomes should be considered preliminary until confirmed in controlled clinical studies.

### 4.6. Inflammatory dermatoses (atopic dermatitis, psoriasis)

Inflammatory dermatoses, including atopic dermatitis (AD) and psoriasis, are characterised by complex immune dysregulation, chronic cutaneous inflammation, and impaired epidermal barrier function. Although current therapies such as corticosteroids, calcineurin inhibitors, and biologics provide symptomatic control, their long-term use is often limited by adverse effects, cost, and incomplete disease resolution. In this context, exosome-based approaches have emerged as promising cell-free strategies with immunomodulatory potential.

#### 4.6.1. Mechanisms of action

Exogenously administered mesenchymal stem cell (MSC)- and adipose-derived stem cell (ADSC)-derived exosomes exert their effects primarily through modulation of immune responses and restoration of epidermal homeostasis. As outlined in the general mechanistic sections, exosomes regulate key inflammatory pathways, including suppression of NF-κB signaling, reduction of pro-inflammatory cytokines, and enhancement of regulatory immune networks. In AD, exosomes have been shown to suppress Th2-mediated responses by downregulating cytokine receptor expression (IL-4Rα, IL-13Rα1, IL-31Rα) and reducing mast cell infiltration, while simultaneously enhancing epidermal barrier protein and lipid synthesis [52,53]. In psoriasis, which is driven predominantly by Th17/Th1 pathways, exosomes attenuate cytokines such as IL-17A, IL-23, and TNF-α and modulate keratinocyte hyperproliferation. In addition to cytokine modulation, exosomes influence immune-cell phenotypes. Increased FOXP3+ regulatory T cells and IL-10 production, along with suppression of pathogenic T-cell responses, have been reported [52,53]. Exosomes also promote macrophage polarization toward an anti-inflammatory M2 phenotype, contributing to resolution of chronic inflammation. At the epidermal level, ADSC-derived exosomes restore keratinocyte homeostasis by reducing oxidative stress and enhancing autophagy-related pathways, including ATG5, Beclin-1, and LC3 expression [54]. These combined effects contribute to improved barrier integrity and reduced susceptibility to inflammatory damage.

#### 4.6.2. Preclinical evidence

Preclinical studies provide substantial evidence supporting the therapeutic potential of exosomes in inflammatory dermatoses. In murine models of AD, IFN-γ-primed MSC-derived exosomes significantly reduced inflammatory cell infiltration, suppressed Th2 signalling pathways, and improved epidermal barrier function, leading to marked improvement in AD-like lesions [52,53]. Similarly, in imiquimod-induced psoriasis models, exosomes derived from human umbilical cord MSCs (hUCMSCs) and adipose-derived MSCs (hADMSCs) reduced epidermal hyperplasia, decreased infiltration of T cells, macrophages, mast cells, and neutrophils, and downregulated pro-inflammatory cytokines and chemokines [55]. Additional studies demonstrate that ADSC-derived exosomes mitigate oxidative stress–induced inflammation in keratinocytes and restore autophagy, further supporting their role in maintaining epidermal homeostasis [54]. Collectively, these findings indicate that exosomes can modulate both immune and epidermal components of inflammatory dermatoses in preclinical settings.

#### 4.6.3. Clinical and translational considerations

Despite robust preclinical evidence, clinical data on exosome therapy in AD and psoriasis remain limited. Current findings are largely derived from *in vitro* and animal studies, and well-designed clinical trials are required to establish efficacy, optimal dosing, delivery methods, and long-term safety. Exosome-based therapies may offer advantages over conventional treatments, including the potential to act as steroid-sparing agents without inducing skin atrophy or systemic adverse effects [55]. However, these benefits remain to be validated in clinical settings. Furthermore, significant challenges persist, including heterogeneity in exosome source, variability in isolation and characterisation methods, and a lack of standardised therapeutic protocols. These limitations currently restrict direct clinical translation and highlight the need for rigorous standardisation and regulatory oversight [56].

### 4.7. Pigment disorders (vitiligo, melasma)

Pigment disorders such as melasma (hyperpigmentation) and vitiligo (depigmentation) arise from dysregulated melanogenesis, oxidative stress, altered keratinocyte-melanocyte signalling, immune-mediated melanocyte damage, and changes in the dermal microenvironment. Exosomes have emerged as key mediators in these processes, exerting both pathological (endogenous) and therapeutic (exogenous) effects depending on their cellular origin and molecular cargo [2].

#### 4.7.1. Vitiligo

Vitiligo is an autoimmune disorder characterised by selective destruction of melanocytes, leading to well-defined depigmented patches [57]. It affects approximately 0.5% of the global population and presents clinically as chronic, milk-white lesions of the skin. Although typically asymptomatic, vitiligo can significantly impair quality of life, contributing to psychological distress, reduced self-esteem, and social anxiety [58].

#### 4.7.2. Mechanisms

Endogenous exosomes derived from keratinocytes under oxidative stress conditions contribute to disease progression by transferring pro-inflammatory microRNAs that activate cytotoxic T cells and exacerbate melanocyte injury [59]. These findings highlight the role of exosomes as mediators of immune-driven melanocyte destruction. Conversely, exogenously administered exosomes, particularly those derived from mesenchymal stem cells (MSCs), exhibit protective and immunomodulatory effects. These vesicles suppress inflammatory signalling, enhance regulatory T-cell (Treg) responses, and reduce oxidative stress in melanocytes [2,60]. In addition, exosomal cargo, including regulatory miRNAs and growth factors, supports melanocyte survival by reducing apoptosis, restoring autophagy, and improving mitochondrial function [50,60].

#### 4.7.3. Preclinical evidence

In a vitiligo model, 3D-culture human umbilical cord MSC-derived exosomes (3D-Exos) were shown to reduce depigmentation through enhancement of Treg-mediated immunosuppression and attenuation of oxidative stress, indicating a dual immunomodulatory and cytoprotective role [60]. These findings support the potential of exosome-based strategies in restoring melanocyte homeostasis.

#### 4.7.4. Clinical and translational considerations

Despite encouraging preclinical data, clinical evidence remains limited. The observed effects should therefore be interpreted as potential therapeutic benefits, and further clinical studies are required to establish long-term efficacy, optimal delivery strategies, and durability of repigmentation.

#### 4.7.5. Melasma

Melasma is a chronic, acquired disorder of hypermelanosis characterised by increased melanin production and deposition in sun-exposed areas of the skin [60,61]. It is influenced by hormonal factors and ultraviolet radiation, while its persistence and recurrence are associated with impaired cellular turnover and dysregulated melanogenesis [62,63].

#### 4.7.6. Mechanisms

Exosomes regulate melanocyte activity through the delivery of signalling molecules that influence melanogenesis and dermal microenvironmental stability. Therapeutic exosomes derived from MSCs and fibroblasts may suppress excessive melanogenesis and improve pigment distribution through modulation of melanogenic pathways and oxidative stress [2]. In addition, exosomes contribute to dermal remodelling by enhancing fibroblast activity, collagen synthesis, and extracellular matrix (ECM) organisation, thereby improving the structural support of melanocyte–keratinocyte units [50]. This indirect effect on the dermal microenvironment may influence pigment stability and overall skin appearance. Exosomal cargo can also directly regulate melanocyte function. For example, certain exosomal miRNAs promote melanogenesis *via* β-catenin-MITF signalling, whereas others suppress tyrosinase activity or induce melanocyte damage depending on their origin and context [64,65]. These findings illustrate the dual regulatory role of exosomes in pigment production.

#### 4.7.7. Preclinical evidence

Preclinical studies suggest that exosomes can modulate melanogenic signalling pathways and improve skin quality through combined effects on melanocyte biology and dermal remodelling. Additionally, exosome-mediated angiogenesis and microenvironmental modulation, *via* pathways such as HIF-1α/VEGF, may enhance dermal perfusion and support tissue regeneration [66,67].

#### 4.7.8. Clinical and translational considerations

Clinical and translational reports of exosome-based approaches for facial pigmentation have noted improvements in skin texture and dermal quality, which may be associated with enhanced ECM organisation and microenvironmental support [68]. However, these findings remain preliminary, and exosome therapy for melasma should be considered investigational, pending validation in controlled clinical studies.

#### 4.7.9. Therapeutic vs pathological roles of exosomes

A key feature of pigmentary disorders is the dual role of exosomes. Endogenous exosomes, particularly those released under oxidative stress or inflammatory conditions, may contribute to melanocyte dysfunction and disease progression, as observed in vitiligo [59]. In contrast, exogenously administered exosomes, especially those derived from MSCs, are being explored for their ability to restore melanocyte homeostasis, regulate immune responses, and improve dermal microenvironmental conditions [2,60]. Understanding this distinction is essential for interpreting experimental findings and for the development of targeted exosome-based therapies in pigmentary disorders.

### 4.8. Acne and scarring

Acne vulgaris is a highly prevalent chronic inflammatory skin disorder affecting adolescents and young adults, with approximately 85% of individuals experiencing mild disease and 15% developing severe forms [69]. Globally, it affects nearly 9% of the population [70]. Its pathogenesis involves four principal factors: excessive sebum production, Cutibacterium acnes proliferation, follicular hyperkeratinisation, and inflammation [71]. Acne scarring represents a frequent and persistent complication, manifesting as either hypertrophic/keloid scars or atrophic scars (icepick, rolling, and boxcar types), often leading to significant psychosocial burden [72,73]. In addition, post-inflammatory hyperpigmentation (PIH) commonly follows acne lesions, particularly in individuals with skin of colour due to increased melanosome size and density [74–77]. Exosome-based therapies have emerged as promising cell-free regenerative approaches, targeting multiple pathogenic pathways involved in inflammation, tissue remodelling, and regeneration.

#### 4.8.1. Mechanisms of action

Exogenously administered exosomes, particularly those derived from mesenchymal stem cells (MSCs) and adipose-derived stem cells (ADSCs), modulate several key processes involved in acne and scar formation. As outlined in earlier sections, these include suppression of inflammatory signalling, regulation of extracellular matrix (ECM) remodelling, enhancement of cellular regeneration, and promotion of angiogenesis. Exosomes attenuate inflammation by downregulating pro-inflammatory cytokines such as IL-1β, TNF-α, and IL-17, while promoting anti-inflammatory signalling pathways and reducing immune-cell infiltration [78,79]. In parallel, they support fibroblast proliferation and collagen synthesis, contributing to balanced ECM remodelling and improved dermal architecture [80–82]. Exosomes also deliver bioactive cargo, including miRNAs, proteins, and lipids, that regulate keratinocyte and fibroblast activity through signalling pathways such as PI3K/AKT and MAPK, thereby enhancing re-epithelialization and tissue repair [83,84]. Additionally, they promote angiogenesis *via* activation of vascular pathways such as AKT/eNOS and VEGF signalling, improving dermal perfusion and supporting regenerative healing [85,86]. Although not the primary target in acne scarring, exosomes may also influence melanocyte activity through transfer of regulatory miRNAs, which could contribute to modulation of post-inflammatory hyperpigmentation [64,87].

#### 4.8.2. Preclinical evidence

Preclinical studies demonstrate that exosomes enhance wound healing and tissue regeneration through coordinated effects on inflammation, fibroblast activity, and angiogenesis. MSC-derived exosomes promote fibroblast proliferation, increase collagen deposition, and improve ECM organisation in injured skin, leading to improved tissue integrity and reduced scar formation [80,81]. Additionally, exosome-mediated activation of regenerative signalling pathways supports keratinocyte proliferation and accelerates re-epithelialization, while modulation of immune responses reduces excessive inflammation that contributes to scar formation [79,83,84]. Exosome-induced angiogenesis further supports tissue repair by enhancing endothelial cell proliferation and neovascularisation, improving oxygen and nutrient delivery to healing tissues [85,86].

#### 4.8.3. Clinical and translational evidence

Emerging clinical evidence supports the therapeutic potential of exosomes in acne scar management. In a prospective, randomised split-face clinical trial, topical application of adipose-derived stem cell exosomes following fractional CO_2_ laser treatment significantly reduced post-procedural inflammation, including erythema and induration, and resulted in greater improvement in acne scar severity compared with control treatment [88]. These findings suggest that exosomes may enhance outcomes of existing procedural therapies by creating a favourable microenvironment for healing. However, clinical data remain limited, and further studies are required to establish standardised treatment protocols, optimal delivery methods, and long-term efficacy.

#### 4.8.4. Clinical and translational considerations

Despite promising results, several limitations remain. Current evidence is predominantly derived from preclinical studies and small clinical trials, and variability in exosome source, isolation methods, and dosing strategies presents challenges for reproducibility and clinical translation. Furthermore, while exosome therapy may improve scar remodelling and skin quality, its ability to achieve complete scar resolution remains uncertain. Therefore, exosome-based interventions should currently be considered adjunctive or investigational approaches in acne and scar management.

## 5. Role of exosomes in oncological skin complications

Exosomes have emerged as key mediators in several oncological skin complications, offering both therapeutic and diagnostic potential. In radiation-induced skin damage, adipose-derived stem cell exosomes (ADSC-Exos) alleviate acute radiation dermatitis by modulating inflammation, promoting macrophage polarisation, enhancing hyaluronic acid synthase 1 expression, and activating reparative TGF-β/Smad2/3 signalling, thereby supporting skin regeneration and repair after radiotherapy [6]. Cancer cell-derived exosomal miR-20a-5p inhibits CD8^+^ T-cell function and confers anti‑PD‑1 therapy resistance in triple‑negative breast cancer[89]. Stem cell-derived exosomes also mitigate radiation-induced injury through reduced oxidative stress, decreased pro-inflammatory cytokines, and improved tissue remodelling, illustrating a multi-modal protective role [90].

Cutaneous adverse events from cancer immunotherapies, such as maculopapular rash, psoriasiform and lichenoid eruptions, are frequent and can limit treatment tolerance, and although direct studies on exosome modulation of these rashes are still emerging, exosome therapies are being explored for their immunoregulatory effects in oncology patients [91]. Furthermore, tumor-derived exosomes (TDEs) have significant biomarker potential in skin cancers; exosomal proteins, RNAs, and circular RNAs isolated from cutaneous squamous cell carcinoma (cSCC) and other skin malignancies reflect the mutational landscape and disease activity, and may serve as non-invasive diagnostic and prognostic biomarkers [92]. Similarly, melanoma-derived exosomes carry tumor-specific cargo that correlates with immune escape, metastatic potential, and treatment response, indicating their utility in monitoring disease progression and therapeutic outcomes [93]. Taken together, exosomes are not only promising agents for mitigating radiation- and therapy-related skin toxicity but also represent a novel class of biomarkers in oncologic dermatology, though further clinical validation is required to realize their full translational impact.

### 5.1. Radiation-induced skin damage

Radiation-induced skin damage is a frequent and debilitating complication of radiotherapy that involves persistent inflammation, oxidative stress, apoptosis, and fibrotic remodeling of cutaneous tissues. Exosomes, particularly those derived from mesenchymal stem cells (MSCs) or adipose-derived stem cells (ADSCs), have shown significant protective effects in preclinical models of radiation-induced skin injury. MSC- and ADSC-exosomes attenuate radiation-induced inflammation by decreasing pro-inflammatory cytokines such as IL-1β and IL-6 and promoting macrophage polarisation toward anti-inflammatory phenotypes, thereby reducing inflammatory cell infiltration and chronic tissue damage [90]. These exosomes also mitigate oxidative stress, lowering reactive oxygen species levels and protecting skin cells from radiation-triggered DNA and mitochondrial damage *via* activation of cellular antioxidant pathways such as Nrf2/ARE, which enhances expression of enzymes like heme oxygenase-1 and superoxide dismutase [94]. Moreover, ADSC-derived exosomes alleviate radiation dermatitis by upregulating hyaluronic acid synthase 1 and activating the TGF-β/Smad2/3 pathway, promoting extracellular matrix remodelling and enhancing skin regeneration [6]. Stem cell-derived exosomes have also been shown to reduce radiation-associated cellular senescence and modulate reparative pathways such as angiogenesis and tissue regeneration, further supporting keratinocyte survival and re-epithelialization after radiation insult [94]. Collectively, these mechanisms, suppression of inflammation, reduction of oxidative stress and fibrosis, and enhancement of keratinocyte and fibroblast survival, underscore the therapeutic promise of exosome-based strategies for managing radiation-induced skin damage, although clinical translation remains in early stages [90].

### 5.2. Chemotherapy-induced skin toxicity

Chemotherapy-induced skin toxicity results from excessive oxidative stress, mitochondrial dysfunction, and apoptosis in epidermal and dermal cells, leading to dermatitis, ulceration, delayed wound healing, and barrier disruption. Exosomes, particularly those derived from mesenchymal stem cells (MSCs), contain antioxidant enzymes and regulatory RNAs that enhance cellular defense mechanisms against chemotherapy-induced oxidative injury. Experimental studies demonstrate that MSC-derived exosomes activate the Nrf2/ARE pathway, upregulating endogenous antioxidant enzymes such as heme oxygenase-1 and superoxide dismutase, thereby reducing reactive oxygen species accumulation in stressed skin cells [90,95]. Exosomes have been shown to activate the Nrf2/ARE antioxidant pathway, increasing expression of endogenous antioxidants and reducing ROS generation in keratinocytes and fibroblasts exposed to oxidative stimuli, which is central to mitigating chemical-induced cytotoxicity [96]. Beyond antioxidation, exosomes can reduce apoptosis by delivering bioactive molecules that modulate cell survival pathways in skin cells exposed to injury. In skin wound healing models, MSC-exosomes enhanced keratinocyte proliferation and migration while suppressing apoptosis induced by oxidative stimuli through inhibition of apoptotic factor translocation and regulation of key mediators such as PARP-1 and AIF, demonstrating direct anti-apoptotic effects relevant to chemotherapy-induced cytotoxicity [97]. Furthermore, engineered “decoy exosomes” have been reported to absorb cytotoxic drugs and reduce off-target damage while preserving anticancer efficacy, demonstrating a strategy to attenuate chemotherapy-induced toxicity *via* exosomal modulation of oxidative and apoptotic pathways [98]. Taken together, these antioxidant and anti-apoptotic actions position exosome-based strategies as promising biologics for protecting cutaneous tissues from chemotherapy-induced injury, though direct clinical validation in oncology patients remains an active area for future research.

### 5.3. Immunotherapy-related rashes

Immunotherapy-related rashes are among the most common cutaneous immune-related adverse events (irCAEs) seen in patients receiving immune checkpoint inhibitors, often reflecting underlying dysregulation of T-cell responses and inflammatory signalling in the skin. irCAEs encompass a spectrum of inflammatory dermatitides including maculopapular, psoriasiform, lichenoid, and eczematous eruptions, arising from heightened activation of cytotoxic CD4^+^/CD8^+^ T cells in response to PD-1/PD-L1 or CTLA-4 blockade [99,100]. Exosomes have well-documented immunomodulatory properties in various contexts, capable of influencing T-cell activation, proliferation, and differentiation, and thus can potentially impact Th1/Th2 balance during immune responses. For example, MSC-derived exosomes can suppress excessive immune activation by inhibiting lymphocyte proliferation and promoting regulatory T-cell populations, contributing to restored immune homeostasis [101]. Furthermore, exosomal cargo such as miRNAs and proteins can modulate signalling pathways critical for helper T-cell differentiation, with potential to shift the Th1/Th2 equilibrium toward a balanced state, thereby attenuating exaggerated Th1- or Th2-mediated inflammation that underlies many immunotherapy-induced rashes [102]. Emerging evidence also suggests that regulation of immune checkpoint pathways by exosomes, such as modulation of antigen presentation and T-cell costimulation, may help mitigate cutaneous adverse events by dampening aberrant T-cell activity while preserving antitumor immunity [103]. Collectively, these immunomodulatory capabilities position exosomes as potential adjunctive agents for reducing immunotherapy-related rashes through immune regulation and Th1/Th2 balance restoration, although direct clinical evidence in this context remains to be established.

## 6. Other biotherapeutic strategies

Other biotherapeutic strategies beyond exosome-based approaches include cell-derived secretomes, growth factor modulation, peptides, and recombinant biologics, all of which aim to directly enhance tissue regeneration, immune regulation, and repair mechanisms in skin disorders and oncologic skin complications [104]. Mesenchymal stem cell secretomes and conditioned media have shown potential to accelerate wound closure, stimulate re-epithelialization, modulate inflammatory responses, and improve scar outcomes in preclinical models, highlighting their promise as cell-free regenerative agents in dermatological applications [105]. In addition, therapeutic peptides play an important role in the field of biopharmaceutics. Recently, various therapeutic peptides have been used in the field of medical research and have great potential in the design of therapeutic schedules [106]. In general, biologic therapies target one extracellular receptor subunit or cytokine, leading to more precise targeting than immunosuppressants and oral JAKi, leading to exceptional long-term safety and no required laboratory monitoring [107].

### 6.1. Stem-cell-based therapies

Mesenchymal stem cells (MSCs) exert much of their therapeutic effect not through engraftment but *via* secretion of a complex secretome comprising cytokines, growth factors, microRNAs, and extracellular vesicles that modulate inflammation, angiogenesis, and tissue repair, making acellular MSC‑derived products promising in regenerative medicine across respiratory, hepatic, and neurological diseases [108]. Adipose‑derived stem cells (ADSCs), a widely accessible MSC source, produce a secretome rich in bioactive factors and exosomes, which enhances wound healing, angiogenesis, immunomodulation, and tissue regeneration with reduced safety concerns compared with cell transplant approaches [109,110]. Induced pluripotent stem cell (iPSC)‑derived MSC secretomes similarly contain pro‑angiogenic and immunosuppressive factors (e.g. MCP‑1/CCL2, IL‑6, IL‑8, angiogenin) that support endothelial repair and suppress inflammatory signalling, highlighting their potential as a standardised and expandable source for cell‑free regenerative interventions [111].

### 6.2. Peptide-based treatments

Bioactive peptides such as signal peptides, copper peptides, and other regenerative peptide sequences are increasingly incorporated into dermatological and cosmetic formulations due to their ability to enhance collagen synthesis, stimulate fibroblast proliferation, and improve skin repair and texture [112]. Signal peptides like palmitoyl pentapeptide-4 and related matrikines trigger signalling cascades that upregulate extracellular matrix components (collagen, elastin, glycosaminoglycans) and have been used in clinical cosmetic products to improve skin elasticity and reduce wrinkle depth [113]. Copper Tripeptide-1 stimulates the synthesis of collagen, elastin, and glycosaminoglycans in the skin, which helps reduce wrinkles and hyperpigmentation while accelerating wound healing. In a rat wound model, administration of Copper Tripeptide-1 increased extracellular matrix deposition. Although its most well-documented effects are skin rejuvenation and wound healing, it also stimulates hair growth and can be used to improve outcomes in hair transplantation procedures. Additionally, Copper Tripeptide-1 exhibits significant antioxidant effects, contributing to the skin’s antioxidant defence by promoting superoxide dismutase activity, neutralising toxic lipid peroxidation products, and modulating the expression of antioxidant-related genes [114].

Additionally, short regenerative peptides designed for enhanced stability and targeted bioactivity have shown promise in promotive wound healing and barrier restoration, suggesting expanding clinical applications beyond traditional cosmetics into therapeutic [115].

### 6.3. Recombinant biologics

Recombinant biologics such as immune checkpoint inhibitors (anti-PD-1/PD-L1 and anti-CTLA-4 antibodies) have revolutionised cancer therapy by blocking inhibitory signalling pathways to enhance anti-tumour T-cell responses, demonstrating significant survival benefit across a range of advanced malignancies, including melanoma and non-small cell lung cancer. Despite their efficacy, use of checkpoint inhibitors frequently induces cutaneous immune-related adverse events including pruritus, rash and vitiligo, underscoring the need for dermatologic management alongside oncologic treatment [116].

In dermatology, monoclonal antibodies such as dupilumab, an IL-4Rα blocker, have become standard of care for moderate-to-severe atopic dermatitis with consistent long-term efficacy and acceptable safety profiles [117]. The primary function of secukinumab is suppressing the IL-17/23 pathway, which is the central driver of psoriatic inflammation. However, the case illustrates that its function is pathway-specific. When faced with a multimorbid condition where psoriasis coexists with a dominant type 2 inflammatory disease like atopic dermatitis, secukinumab’s activity is insufficient to control the concurrent type 2 (IL-4/IL-13 driven) inflammation, leading to suboptimal clinical outcomes when used as monotherapy [118].

Recombinant biologics represent a class of precision immunotherapies whose clinical efficacy in skin cancers and inflammatory diseases stems from their targeted modulation of specific molecular pathways [117].

## 7. Comparative analysis of exosomes VS. other biotherapeutics

Exosomes offer multimodal, cell-derived signalling with low immunogenic potential and attractive cell-free regenerative properties, but clinical durability, standardised manufacturing, and clarity on safety remain evolving [119]. Other biotherapeutics (growth factors, peptides, monoclonals, checkpoint inhibitors) deliver predictable, target-specific effects with mature manufacturing and safety datasets, but can produce well-defined systemic toxicities and often address single mechanistic nodes rather than broad regenerative programs [120]. A comparative analysis of exosomes and other biotherapeutics is shown in Table 1.

**Table 1.** Comparative analysis of exosomes vs. other biotherapeutics.

Category	Exosomes	Other biotherapeutics	References
Mechanism	Primarily act as paracrine mediators: deliver proteins, miRNAs and lipids to reprogram recipient cells (immunomodulation, angiogenesis, ECM remodeling).	Act through defined single/multi-molecule mechanisms: receptor blockade (mAbs), cytokine neutralization or growth-factor receptor stimulation, typically a single, high-affinity molecular target.	[119,121]
Safety	Preclinical and early clinical reports suggest generally mild local reactions; safety concerns focus on cargo heterogeneity, potential off-target signaling, and contamination if poorly manufactured.	Well-characterized safety profiles in large trials for many agents (e.g., dupilumab, secukinumab); class toxicities are known (infection risk, systemic immunomodulation, checkpoint-related irAEs).	[122,123]
Duration of effect	Effects often transient after single local dosing; repeated or engineered/encapsulated exosomes can prolong response but clinical durability data remain limited.	Many biologics produce sustained, predictable clinical responses with defined dosing intervals (weeks–months) and well-documented long-term efficacy	[121,124]
Manufacturing challenges	Major hurdles: standardized isolation, yield, potency assays, batch variability, sterile GMP production and cold-chain storage; scalable EV manufacturing is emerging but not yet routine	Mature large-scale manufacturing platforms exist for mAbs and recombinant proteins with robust process controls, regulatory pathways and well-established cold-chain/formulation technologies.	[119,120,125]
Limitations (stability, dosing, delivery routes)	Stability: sensitive to storage/processing; dosing poorly defined; delivery: topical/intradermal routes promising but biodistribution and clearance are variable.	Stability/formulation: many biologics are stable in validated formulations; dosing regimens standardized; delivery mainly systemic or targeted local injections, but systemic delivery risks systemic adverse events.	[123,126,127]

## 8. Exosome production, isolation, and therapeutic preparation

The development of exosome-based therapies requires standardised and reproducible approaches for exosome production, isolation, and characterisation, which remain key challenges in clinical translation. Therapeutic exosomes are commonly derived from mesenchymal stem cells (MSCs), adipose-derived stem cells (ADSCs), umbilical cord-derived cells, or dermal fibroblasts cultured under controlled *in vitro* conditions. These sources are selected for their high exosome yield and favourable regenerative and immunomodulatory profiles. Culture conditions, including hypoxia and inflammatory priming, can significantly influence exosome cargo composition and functional activity. Isolation of exosomes from conditioned media or biological fluids is typically performed using differential ultracentrifugation, size-exclusion chromatography, polymer-based precipitation, or immunoaffinity-based methods targeting surface markers such as CD63 and CD81[119]. Each technique presents specific advantages and limitations in terms of purity, yield, scalability, and cost, contributing to variability across studies. For therapeutic applications, exosomes may be derived from autologous or allogeneic sources, with each approach offering distinct advantages in terms of immunogenicity and scalability. Additionally, engineered exosomes are being developed through cargo loading and surface modification strategies to enhance targeting and therapeutic efficacy [126]. Standardised characterisation, including particle size distribution, molecular cargo profiling, and functional assays, is essential to ensure reproducibility, potency, and safety of exosome-based therapeutics.

## 9. Safety considerations and potential risks of exosome therapy

Despite their promising therapeutic potential, exosome-based interventions present several safety concerns that must be addressed for clinical application. One major concern is the potential for pro-tumorigenic effects, particularly when exosomes are derived from inappropriate or insufficiently characterised sources. Tumour-derived or contaminated exosomes may promote angiogenesis, immune evasion, and tumour progression [31–33]. Even stem cell-derived exosomes may carry bioactive molecules that could influence tumour-related pathways under certain conditions. Immunogenicity is another consideration, particularly for allogeneic exosomes, which may elicit immune responses depending on their surface protein composition. Additionally, the ability of exosomes to distribute systemically raises concerns regarding off-target effects and unintended modulation of non-target tissues.

Variability in isolation and purification methods may also result in contamination with proteins, lipoproteins, or other extracellular vesicles, potentially affecting both safety and efficacy [119]. Furthermore, dysregulated signalling induced by exosomes may lead to excessive fibrosis or abnormal tissue remodelling. Addressing these challenges requires rigorous source selection, standardised manufacturing protocols, detailed molecular characterisation, and long-term clinical safety evaluation to ensure safe and effective therapeutic application [2].

## 10. Translational and clinical

Clinical translation of exosome-based and related biotherapeutic strategies in dermatology is progressing, with early-phase clinical trials and small clinical series reporting benefits in wound healing, skin rejuvenation, pigmentary disorders, and hair restoration, although large randomised controlled trials remain limited [128].

Systematic reviews emphasise that existing clinical studies are heterogeneous in design, dosing, endpoints, and product characterisation, underscoring the need for standardised protocols and robust efficacy and safety validation before widespread clinical adoption [129]. Beyond their intrinsic biological activity, exosomes are being actively developed as drug-delivery systems, as their native lipid bilayer supports efficient cargo loading, cellular uptake, and relative immune evasion compared with synthetic nanoparticles [126].

Advances in exosome engineering, including optimised loading techniques, surface functionalization, and hybrid biomaterial platforms, aim to improve payload stability, targeting specificity, and tissue retention for therapeutic applications [130]. These regulatory uncertainties are particularly evident in the divergence between cosmetic and medical uses, where exosome-containing products are already marketed and applied off-label in aesthetic practice with variable oversight and limited clinical evidence[131]. In contrast, medical applications for dermatological and oncologic indications require rigorous clinical trials, standardised manufacturing, and regulatory approval to ensure safety, reproducibility, and therapeutic efficacy [132].

## 11. Future directions

Engineered exosomes are being developed to enhance targeting, retention, and therapeutic efficacy by modifying surface ligands or cargo composition, which may improve clinical outcomes in cancer and regenerative medicine [133]. Artificial exosomes or synthetic mimetics generated through top-down and bottom-up nanofabrication hold promise for overcoming natural exosome limitations such as low yield and heterogeneity, potentially enabling more scalable and standardised therapeutic [123]. Hybrid nanovesicles that combine natural exosome membranes with synthetic elements or liposomal structures may offer improved drug loading, controlled release and enhanced biodistribution compared with unmodified exosomes [134]. Personalised exosome therapy aims to tailor exosome source, cargo and delivery based on individual patient characteristics and disease profiles, expanding precision medicine applications across oncology and regenerative indications [135].

Large-scale exosome manufacturing remains a bottleneck for clinical translation, requiring innovations in standardised bioreactor design, high-yield isolation, and quality-controlled, cost-effective processes to generate clinical-grade products. Achieving this will necessitate the development of closed, automated systems with integrated purification to ensure batch-to-batch consistency and meet regulatory demands [136].

## 12. Ethical considerations in future exosome therapies

Ethical considerations for the clinical translation of exosome-based therapies include establishing robust regulatory frameworks that ensure safety, efficacy, and quality control for products derived from living cells, as the biological variability and lack of standardised manufacturing raise ethical concerns around patient risk and oversight [137]. Additionally, exosomes sourced from human tissues necessitate clear informed consent and transparent donor protocols to respect donor autonomy and address moral questions related to the use of human biological material [138]. The potential for direct-to-consumer marketing of unapproved exosome products underscores the need for ethical oversight to prevent unregulated clinical or cosmetic use that may expose patients to harm and exacerbate inequities in access to advanced therapies.

## 13. Summary of evidence

Accumulating preclinical and translational evidence indicates that exosomes derived from mesenchymal stem cells, adipose-derived stem cells, fibroblasts, and other skin-relevant sources exert potent regenerative and immunomodulatory effects across diverse dermatological and oncologic skin conditions. In wound-healing models, exosome therapies consistently enhance re-epithelialization, angiogenesis, collagen deposition, and macrophage polarisation toward reparative phenotypes while suppressing pro-inflammatory signalling, leading to improved tissue architecture and reduced scarring.

In photoaged and UV-damaged skin, exosomes attenuate oxidative stress, suppress matrix degradation, restore extracellular matrix integrity, and reduce cellular senescence through modulation of antioxidant and TGF-β–related pathways. In inflammatory dermatoses such as atopic dermatitis and psoriasis, exosomes downregulate pathogenic Th2/Th17/Th1 responses, inhibit NF-κB activation, enhance regulatory immune pathways, and restore epidermal barrier and autophagy functions, resulting in marked improvement in disease severity in preclinical models.

Exosomes also influence pigmentary disorders by regulating melanocyte survival, melanogenesis, immune-mediated melanocyte injury, and dermal microenvironment support. In oncologic settings, physiological exosomes mitigate radiation- and chemotherapy-induced skin toxicity, whereas tumour-derived exosomes display pro-tumorigenic and immunosuppressive properties, highlighting the critical importance of source selection and molecular characterisation. Despite these advances, clinical translation remains limited by heterogeneity in isolation and characterisation, scalability challenges, uncertain biodistribution, and unresolved long-term safety concerns.

## 14. Conclusion

Exosome-based and allied biotherapeutic strategies offer a promising, cell-free approach for addressing complex dermatological diseases and oncologic therapy–associated skin complications by targeting core mechanisms of inflammation, immune dysregulation, oxidative stress, and impaired regeneration. While preclinical data are compelling, widespread clinical adoption will require standardised manufacturing and characterisation protocols, rigorous safety evaluation, and well-designed clinical trials. Continued innovation in engineered exosomes, targeted delivery systems, and regulatory frameworks is essential to realise the full translational potential of exosome-centred therapies in dermatology and cutaneous oncology.

## Data Availability

Data sharing is not applicable to this review as the article does not involve the creation or analysis of new data.

## References

[1] Hu J-C, Zheng C-X, Sui B-D, et al. Mesenchymal stem cell-derived exosomes: a novel and potential remedy for cutaneous wound healing and regeneration. World J Stem Cells. 2022;14(5):318–329. doi: 10.4252/wjsc.v14.i5.318. 35722196 PMC9157601

[2] Tienda-Vázquez MA, Hanel JM, Márquez-Arteaga EM, et al. Exosomes: a promising strategy for repair, regeneration and treatment of skin disorders. Cells. 2023;12(12):1625. doi: 10.3390/cells12121625. 37371095 PMC10296902

[3] Olumesi KR, Goldberg DJ. A review of exosomes and their application in cutaneous medical aesthetics. J Cosmet Dermatol. 2023;22(10):2628–2634. doi: 10.1111/jocd.15930. 37498301

[4] Phinney DG, Pittenger MF. Concise review: MSC-derived exosomes for cell-free Therapy. Stem Cells. 2017;35(4):851–858. doi: 10.1002/stem.2575. 28294454

[5] Mahmoud RH, Peterson E, Badiavas EV, et al. Exosomes: a comprehensive review for the practicing dermatologist. J Clin Aesthet Dermatol. 2025;18(4):33–40. PMC12007658 40256340

[6] Li M, Tian Y, Wang X, et al. Exosomes derived from adipose-derived stem cells alleviate acute radiation-induced dermatitis through up-regulating hyaluronic acid synthase 1 expression. Stem Cell Res Ther. 2025;16(1):253. doi: 10.1186/s13287-025-04276-8. 40394699 PMC12093883

[7] Wang Y, Ding H, Bai R, et al. Exosomes from adipose-derived stem cells accelerate wound healing by increasing the release of IL-33 from macrophages. Stem Cell Res Ther. 2025;16(1):80. doi: 10.1186/s13287-025-04203-x. 39984984 PMC11846291

[8] Kalluri R, LeBleu VS. The biology, function, and biomedical applications of exosomes. Science. 2020;367(6478):eaau6977. doi: 10.1126/science.aau6977. 32029601 PMC7717626

[9] Liese S, Wenzel EM, Kjos I, et al. Protein crowding mediates membrane remodeling in upstream ESCRT-induced formation of intraluminal vesicles. Proc Natl Acad Sci U S A. 2020;117(46):28614–28624. doi: 10.1073/pnas.2014228117. 33139578 PMC7682568

[10] Pegtel DM, Gould SJ. Exosomes. Annu Rev Biochem. 2019;88(1):487–514. doi: 10.1146/annurev-biochem-013118-111902. 31220978

[11] Hessvik NP, Llorente A. Current knowledge on exosome biogenesis and release. Cell Mol Life Sci. 2018;75(2):193–208. doi: 10.1007/s00018-017-2595-9. 28733901 PMC5756260

[12] Arya SB, Collie SP, Parent CA. The ins-and-outs of exosome biogenesis, secretion, and internalization. Trends Cell Biol. 2024;34(2):90–108. doi: 10.1016/j.tcb.2023.06.006. 37507251 PMC10811273

[13] Tancini B, Buratta S, Delo F, et al. Lysosomal exocytosis: the extracellular role of an intracellular organelle. Membranes (Basel). 2020;10(12): 406. doi: 10.3390/membranes10120406. PMC7764620 33316913

[14] Kuo I-Y, Hsieh C-H, Kuo W-T, et al. Recent advances in conventional and unconventional vesicular secretion pathways in the tumor microenvironment. J Biomed Sci. 2022;29(1):56. doi: 10.1186/s12929-022-00837-8. 35927755 PMC9354273

[15] Lee YJ, Shin KJ, Chae YC. Regulation of cargo selection in exosome biogenesis and its biomedical applications in cancer. Exp Mol Med. 2024;56(4):877–889. doi: 10.1038/s12276-024-01209-y. 38580812 PMC11059157

[16] Ju J, Neuen SML, van Zandvoort M, et al. Extracellular vesicles: cargo loading, degradation and secretory pathways, and their intersection with autophagy. Extracell Vesicles Circ Nucl Acids. 2025;6(3):360–385. doi: 10.20517/evcna.2025.21. 41132500 PMC12540057

[17] Xie S, Zhang Q, Jiang L. Current knowledge on exosome biogenesis, cargo-sorting mechanism and therapeutic implications. Membranes (Basel). 2022;12(5):498. doi: 10.3390/membranes12050498. PMC9144303 35629824

[18] Wang T, Zhang H. Exploring the roles and molecular mechanisms of RNA binding proteins in the sorting of noncoding RNAs into exosomes during tumor progression. J Adv Res. 2024;65:105–123. doi: 10.1016/j.jare.2023.11.029. 38030125 PMC11518959

[19] Chen Y, Zhao Y, Yin Y, et al. Mechanism of cargo sorting into small extracellular vesicles. Bioengineered. 2021;12(1):8186–8201. doi: 10.1080/21655979.2021.1977767. 34661500 PMC8806638

[20] Chen Y-F, Luh F, Ho Y-S, et al. Exosomes: a review of biologic function, diagnostic and targeted therapy applications, and clinical trials. J Biomed Sci. 2024;31(1):67. doi: 10.1186/s12929-024-01055-0. 38992695 PMC11238361

[21] Liu YJ, Wang C. A review of the regulatory mechanisms of extracellular vesicles-mediated intercellular communication. Cell Commun Signal. 2023;21(1):77. doi: 10.1186/s12964-023-01103-6. 37055761 PMC10100201

[22] Xu Y-P, Jiang T, Yang X-F, et al. Methods, mechanisms, and application prospects for enhancing extracellular vesicle uptake. Curr Med Sci. 2024;44(2):247–260. doi: 10.1007/s11596-024-2861-7. 38622425

[23] Wu D, Sun H, Yang B, et al. Exosome heterogeneity affects the distal “barrier-crossing” trafficking of exosome encapsulated quantum dots. ACS Nano. 2024;18(11):7907–7922. doi: 10.1021/acsnano.3c09378. 38394382

[24] Schmidtmann M, D’Souza-Schorey C. Extracellular vesicles: biological packages that modulate tumor cell invasion. Cancers (Basel). 2023;15(23):5617. doi: 10.3390/cancers15235617. 38067320 PMC10705367

[25] Avalos PN, Forsthoefel DJ. An emerging frontier in intercellular communication: extracellular vesicles in regeneration. Front Cell Dev Biol. 2022;10:849905. doi: 10.3389/fcell.2022.849905. 35646926 PMC9130466

[26] Hoshino A, Kim HS, Bojmar L, et al. Extracellular vesicle and particle biomarkers define multiple human cancers. Cell. 2020;182(4):1044–1061.e18. doi: 10.1016/j.cell.2020.07.009. 32795414 PMC7522766

[27] Padinharayil H, Varghese J, Wilson C, et al. Mesenchymal stem cell-derived exosomes: characteristics and applications in disease pathology and management. Life Sci. 2024;342:122542. doi: 10.1016/j.lfs.2024.122542. 38428567

[28] Wei B, Wei M, Huang H, et al. Mesenchymal stem cell-derived exosomes: a promising therapeutic strategy for age-related diseases. Cell Prolif. 2025;58(5):e13795. doi: 10.1111/cpr.13795. 39704104 PMC12099225

[29] Zhao B, Wei J, Jiang Z, et al. Mesenchymal stem cell-derived exosomes: an emerging therapeutic strategy for hepatic ischemia-reperfusion injury. Stem Cell Res Ther. 2025;16(1):178. doi: 10.1186/s13287-025-04302-9. 40229893 PMC11998454

[30] Ahmadpour F, Rasouli HR, Talebi S, et al. Effects of exosomes derived from fibroblast cells on skin wound healing in Wistar rats. Burns. 2023;49(6):1372–1381. doi: 10.1016/j.burns.2023.02.003. 36828692

[31] Javdani-Mallak A, Mowla SJ, Alibolandi M. Tumor-derived exosomes and their application in cancer treatment. J Transl Med. 2025;23(1):751. doi: 10.1186/s12967-025-06814-7. 40629355 PMC12236016

[32] Bai S, Wang Z, Wang M, et al. Tumor-derived exosomes modulate primary site tumor metastasis. Front Cell Dev Biol. 2022;10:752818. doi: 10.3389/fcell.2022.752818. 35309949 PMC8924426

[33] Li J, Yu S, Rao M, et al. Tumor-derived extracellular vesicles: key drivers of immunomodulation in breast cancer. Front Immunol. 2025;16:1548535. doi: 10.3389/fimmu.2025.1548535. 40103824 PMC11914124

[34] Yu H, Feng H, Zeng H, et al. Exosomes: the emerging mechanisms and potential clinical applications in dermatology. Int J Biol Sci. 2024;20(5):1778–1795. doi: 10.7150/ijbs.92897. 38481799 PMC10929203

[35] Norouzi F, Aghajani S, Vosoughi N, et al. Exosomes derived stem cells as a modern therapeutic approach for skin rejuvenation and hair regrowth. Regen Ther. 2024;26:1124–1137. doi: 10.1016/j.reth.2024.10.001. 39640923 PMC11617408

[36] Li W, Pang Y, He Q, et al. Exosome-derived microRNAs: emerging players in vitiligo. Front Immunol. 2024;15:1419660. doi: 10.3389/fimmu.2024.1419660. 39040109 PMC11260631

[37] Zhang R, Wei Y, Wang T, et al. Exosomal miRNAs in autoimmune skin diseases. Front Immunol. 2023;14:1307455. doi: 10.3389/fimmu.2023.1307455. 38106405 PMC10722155

[38] Calabrese EJ, Dhawan G, Kapoor R, et al. Hormesis: wound healing and keratinocytes. Pharmacol Res. 2022;183:106393. doi: 10.1016/j.phrs.2022.106393. 35961478

[39] Jo H, Brito S, Kwak BM, et al. Applications of mesenchymal stem cells in skin regeneration and rejuvenation. Int J Mol Sci. 2021;22(5):2410. doi: 10.3390/ijms22052410. 33673711 PMC7957487

[40] Surowiecka A, Strużyna J. Adipose-derived stem cells for facial rejuvenation. J Pers Med. 2022;12(1):117. doi: 10.3390/jpm12010117. 35055432 PMC8781097

[41] Zhou L, Wang H, Yao S, et al. Efficacy of human adipose derived mesenchymal stem cells in promoting skin wound healing. J Healthc Eng. 2022;2022:6590025–6590025. doi: 10.1155/2022/6590025. 35368914 PMC8970852

[42] Guillamat-Prats R. The role of MSC in wound healing, scarring and regeneration. Cells. 2021;10(7):1729. doi: 10.3390/cells10071729. 34359898 PMC8305394

[43] Mazini L, Rochette L, Admou B, et al. Hopes and limits of adipose-derived stem cells (ADSCs) and mesenchymal stem cells (MSCs) in wound healing. Int J Mol Sci. 2020;21(4):1306. doi: 10.3390/ijms21041306. 32075181 PMC7072889

[44] Bian D, Wu Y, Song G, et al. The application of mesenchymal stromal cells (MSCs) and their derivative exosome in skin wound healing: a comprehensive review. Stem Cell Res Ther. 2022;13(1):24. doi: 10.1186/s13287-021-02697-9. 35073970 PMC8785459

[45] Song Y, You Y, Xu X, et al. Adipose-derived mesenchymal stem cell-derived exosomes biopotentiated extracellular matrix hydrogels accelerate diabetic wound healing and skin regeneration. Adv Sci (Weinh). 2023;10(30):e2304023. doi: 10.1002/advs.202304023. 37712174 PMC10602544

[46] Wang AS, Dreesen O. Biomarkers of cellular senescence and skin aging. Front Genet. 2018;9:247. doi: 10.3389/fgene.2018.00247. 30190724 PMC6115505

[47] Csekes E, Račková L. Skin aging, cellular senescence and natural polyphenols. Int J Mol Sci. 2021;22(23):12641. doi: 10.3390/ijms222312641. 34884444 PMC8657738

[48] Tanveer MA, Rashid H, Tasduq SA. Molecular basis of skin photoaging and therapeutic interventions by plant-derived natural product ingredients: a comprehensive review. Heliyon. 2023;9(3):e13580. doi: 10.1016/j.heliyon.2023.e13580. 36895391 PMC9988502

[49] Hajialiasgary Najafabadi A, Soheilifar MH, Masoudi-Khoram N. Exosomes in skin photoaging: biological functions and therapeutic opportunity. Cell Commun Signal. 2024;22(1):32. doi: 10.1186/s12964-023-01451-3. 38217034 PMC10785444

[50] Park AY, Lee JO, Jang Y, et al. Exosomes derived from human dermal fibroblasts protect against UVB‑induced skin photoaging. Int J Mol Med. 2023;52(6) :120. doi: 10.3892/ijmm.2023.5323. PMC10635689 37888610

[51] He C, Wang Z, Jiang Z, et al. Microneedles combining delivery of hUMSC-derived exosomes and EGCG mitigate UV-induced skin damage. J Nanobiotechnology. 2025;23(1):643. doi: 10.1186/s12951-025-03735-x. 41074154 PMC12514848

[52] Huang YC, Chang CY, Huang CJ. Effectiveness of exosomes from different mesenchymal stem cells in the treatment of psoriasis: a murine study and meta-analysis of experimental studies. Biomedicines. 2025;13(9):2093. doi: 10.3390/biomedicines13092093. 41007656 PMC12467663

[53] Chen Y, Liu H, He Y, et al. Roles for exosomes in the pathogenesis, drug delivery and therapy of psoriasis. Pharmaceutics. 2025;17(1):51. doi: 10.3390/pharmaceutics17010051. 39861699 PMC11768235

[54] Kim HR, Lee SY, You GE, et al. Adipose-derived stem cell exosomes alleviate psoriasis serum exosomes-induced inflammation by regulating autophagy and redox status in keratinocytes. Clin Cosmet Investig Dermatol. 2023;16:3699–3711. doi: 10.2147/CCID.S439760. PMC10752035 38152151

[55] Siriphanit R, Thanasarnaksorn W, Boonpethkaew S, et al. Exosome-based therapy: a comparative study of adipose- and umbilical cord-derived mesenchymal stem cells-derived exosomes in psoriatic mouse model. Exp Dermatol. 2025;34(11):e70176. doi: 10.1111/exd.70176. 41217063

[56] He K, Zang J, Ren T, et al. Therapeutic potential and mechanisms of mesenchymal stem cell and mesenchymal stem cell-derived extracellular vesicles in atopic dermatitis. J Inflamm Res. 2024;17:5783–5800. doi: 10.2147/JIR.S479444. 39224661 PMC11368146

[57] Frisoli ML, Essien K, Harris JE. Vitiligo: mechanisms of pathogenesis and treatment. Annu Rev Immunol. 2020;38(1):621–648. doi: 10.1146/annurev-immunol-100919-023531. 32017656

[58] Dabas G, Vinay K, Parsad D, et al. Psychological disturbances in patients with pigmentary disorders: a cross-sectional study. J Eur Acad Dermatol Venereol. 2020;34(2):392–399. doi: 10.1111/jdv.15987. 31566833

[59] Ma J, Zhou Y, Chen J, et al. Exosomes enriched with miR-31-3p from keratinocytes under oxidative stress promote vitiligo progression by destructing melanocytes and activating CD8(+) T cells. Int J Biol Macromol. 2025;310(Pt 1):143070. doi: 10.1016/j.ijbiomac.2025.143070. 40220810

[60] Wang Q, Guo W, Niu L, et al. 3D-hUMSCs exosomes ameliorate vitiligo by simultaneously potentiating treg cells-mediated immunosuppression and suppressing oxidative stress-induced melanocyte damage. Adv Sci (Weinh). 2024;11(31):e2404064. doi: 10.1002/advs.202404064. 38887870 PMC11336971

[61] Espósito ACC, Cassiano DP, da Silva CN, et al. Update on melasma-part I: pathogenesis. Dermatol Ther (Heidelb). 2022;12(9):1967–1988. doi: 10.1007/s13555-022-00779-x. 35904706 PMC9464278

[62] Hughes BK, Bishop CL. Current Understanding of the role of senescent melanocytes in skin ageing. Biomedicines. 2022;10(12):3111. doi: 10.3390/biomedicines10123111. 36551868 PMC9775966

[63] Kang HY, Lee JW, Papaccio F, et al. Alterations of the pigmentation system in the aging process. Pigment Cell Melanoma Res. 2021;34(4):800–813. doi: 10.1111/pcmr.12994. 34048137

[64] Zhao C, Wang D, Wang X, et al. Down-regulation of exosomal miR-200c derived from keratinocytes in vitiligo lesions suppresses melanogenesis. J Cell Mol Med. 2020;24(20):12164–12175. doi: 10.1111/jcmm.15864. 32918341 PMC7579706

[65] Yoon JH, Jo CS, Hwang JS. Comprehensive analysis of exosomal MicroRNAs derived from UVB-irradiated keratinocytes as potential melanogenesis regulators. Int J Mol Sci. 2024;25(6):3095. doi: 10.3390/ijms25063095. 38542076 PMC10970293

[66] Luo G, Zhou Z, Cao Z, et al. M2 macrophage-derived exosomes induce angiogenesis and increase skin flap survival through HIF1AN/HIF-1α/VEGFA control. Arch Biochem Biophys. 2024;751:109822. doi: 10.1016/j.abb.2023.109822. 38030054

[67] Chen Y, Yin W, Liu Z, et al. Exosomes derived from fibroblasts enhance skin wound angiogenesis by regulating HIF-1α/VEGF/VEGFR pathway. Burns Trauma. 2025;13:tkae071. doi: 10.1093/burnst/tkae071. 40433567 PMC12107542

[68] Wang T, Gao H, Wang D, et al. Stem cell-derived exosomes in the treatment of melasma and its percutaneous penetration. Lasers Surg Med. 2023;55(2):178–189. doi: 10.1002/lsm.23628. 36573453

[69] Ryguła I, Pikiewicz W, Kaminiów K. Impact of diet and nutrition in patients with acne vulgaris. Nutrients. 2024;16(10):1476. doi: 10.3390/nu16101476. 38794714 PMC11124289

[70] Forraz N, Bize C, Desroches A-L, et al. The world’s first acne dysbiosis-like model of human 3D Ex vivo sebaceous gland colonized with cutibacterium acnes and staphylococcus epidermidis. Microorganisms. 2023;11(9):2183. doi: 10.3390/microorganisms11092183. 37764027 PMC10537848

[71] Baldwin H, Tan J. Effects of diet on acne and its response to treatment. Am J Clin Dermatol. 2021;22(1):55–65. doi: 10.1007/s40257-020-00542-y. 32748305 PMC7847434

[72] Tan J, Beissert S, Cook-Bolden F, et al. Impact of facial atrophic acne scars on quality of life: a multi-country population-based survey. Am J Clin Dermatol. 2022;23(1):115–123. doi: 10.1007/s40257-021-00628-1. 34705166 PMC8776674

[73] Liu L, Xue Y, Chen Y, et al. Prevalence and risk factors of acne scars in patients with acne vulgaris. Skin Res Technol. 2023;29(6):e13386. doi: 10.1111/srt.13386. 37357642 PMC10240192

[74] Auffret N, Leccia M-T, Ballanger F, et al. Acne-induced post-inflammatory hyperpigmentation: from grading to treatment. Acta Derm Venereol. 2025;105:adv42925. doi: 10.2340/actadv.v105.42925. 40263971 PMC12041799

[75] Anvery N, Christensen RE, Dirr MA. Management of post-inflammatory hyperpigmentation in skin of color: a short review. J Cosmet Dermatol. 2022;21(5):1837–1840. doi: 10.1111/jocd.14916. 35289059

[76] Sangha AM. Managing post-inflammatory hyperpigmentation in patients with acne. J Clin Aesthet Dermatol. 2021;14(6 Suppl 1)Suppl 1): :S24–s26. PMC8565877 34976295

[77] Tan MG, Kim WB, Jo CE, et al. Topical treatment for postinflammatory hyperpigmentation: a systematic review. J Dermatolog Treat. 2022;33(5):2518–2526. doi: 10.1080/09546634.2021.1981814. 34525885

[78] Lotfy A, AboQuella NM, Wang H. Mesenchymal stromal/stem cell (MSC)-derived exosomes in clinical trials. Stem Cell Res Ther. 2023;14(1):66. doi: 10.1186/s13287-023-03287-7. 37024925 PMC10079493

[79] Ding H, Wang Y, Bai R, et al. Exosomes from adipose-derived stem cells inhibit skin t-cell activation and alleviate wound inflammation. Aesthet Surg J. 2025;45(7):723–734. doi: 10.1093/asj/sjaf040. 40100757

[80] Zhao H, Li Z, Wang Y, et al. Bioengineered MSC-derived exosomes in skin wound repair and regeneration. Front Cell Dev Biol. 2023;11:1029671. doi: 10.3389/fcell.2023.1029671. 36923255 PMC10009159

[81] Yan L, Fan D, Yang J, et al. Fibroblast exosomes promote wound healing and improve the quality of healed skin via miR-29a-3p-mediated KEAP1/Nrf2 pathway activation. Burns Trauma. 2025;13:tkaf035. doi: 10.1093/burnst/tkaf035. 41146891 PMC12554163

[82] Kang D, Wang X, Chen W, et al. Epidermal stem cell-derived exosomes improve wound healing by promoting the proliferation and migration of human skin fibroblasts. Burns Trauma. 2024;12:tkae047. doi: 10.1093/burnst/tkae047. 39687464 PMC11647520

[83] Zhou C, Zhang B, Yang Y, et al. Stem cell-derived exosomes: emerging therapeutic opportunities for wound healing. Stem Cell Res Ther. 2023;14(1):107. doi: 10.1186/s13287-023-03345-0. 37101197 PMC10134577

[84] Dawes JS, Abdelaal M, Landmesser ME, et al. Exosomes: the future of acellular nanotherapeutics in regenerative vascularization. Front Bioeng Biotechnol. 2025;13:1607605. doi: 10.3389/fbioe.2025.1607605. 40979643 PMC12443856

[85] Qiu X, Liu J, Zheng C, et al. Exosomes released from educated mesenchymal stem cells accelerate cutaneous wound healing via promoting angiogenesis. Cell Prolif. 2020;53(8):e12830. doi: 10.1111/cpr.12830. 32608556 PMC7445410

[86] Che D, Xiang X, Xie J, et al. Exosomes derived from adipose stem cells enhance angiogenesis in diabetic wound via miR-146a-5p/JAZF1 Axis. Stem Cell Rev Rep. 2024;20(4):1026–1039. doi: 10.1007/s12015-024-10685-8. 38393667 PMC11087353

[87] Zhao H, Jo C, Hwang J. Exosomal miR-365b-5p derived from keratinocyte promotes melanogenesis by directly targeting GLI2. Arch Dermatol Res. 2025;317(1):355. doi: 10.1007/s00403-025-03841-8. 39918780

[88] Kwon HH, Yang SH, Lee J, et al. Combination treatment with human adipose tissue stem cell-derived exosomes and fractional CO2 laser for acne scars: a 12-week prospective, double-blind, randomized, split-face study. Acta Derm Venereol. 2020;100(18):adv00310. doi: 10.2340/00015555-3666. 33073298 PMC9309822

[89] Li W, Han G, Li F, et al. Cancer cell-derived exosomal miR-20a-5p inhibits CD8(+) T-cell function and confers anti-programmed cell death 1 therapy resistance in triple-negative breast cancer. Cancer Sci. 2024;115(2):347–356. doi: 10.1111/cas.16036. 38129137 PMC10859600

[90] Liu Z, Gu J, Gao Y, et al. ADSC-derived exosomes mitigate radiation-induced skin injury by reducing oxidative stress, inflammation and cell death. Front Public Health. 2025;13:1603431. doi: 10.3389/fpubh.2025.1603431. 40438040 PMC12116344

[91] Nikolaou V, Tsimpidakis A, Stratigos A. Cutaneous adverse reactions of immunotherapy in patients with advanced melanoma. Cancers (Basel). 2023;15(7):2084. doi: 10.3390/cancers15072084. 37046745 PMC10093334

[92] Lee IT-L, Shen C-H, Tsai F-C, et al. Cancer-derived extracellular vesicles as biomarkers for cutaneous squamous cell carcinoma: a systematic review. Cancers (Basel). 2022;14(20):5098. doi: 10.3390/cancers14205098. 36291882 PMC9599948

[93] Alia Moosavian S, Hashemi M, Etemad L, et al. Melanoma-derived exosomes: versatile extracellular vesicles for diagnosis, metastasis, immune modulation, and treatment of melanoma. Int Immunopharmacol. 2022;113(Pt A):109320. doi: 10.1016/j.intimp.2022.109320. 36274482

[94] Kuo PJ, Rau CS, Hsieh CH. Advances in stem cell-derived exosome therapy for radiation-induced skin injury. Int J Surg. 2025;111(11):8336–8348. doi: 10.1097/JS9.0000000000003004. 40844299 PMC12626575

[95] Lei L, Zhou S, Zeng L, et al. Exosome-based therapeutics in dermatology. Biomater Res. 2025;29:0148. doi: 10.34133/bmr.0148. 40351703 PMC12062580

[96] Wang T, Jian Z, Baskys A, et al. MSC-derived exosomes protect against oxidative stress-induced skin injury via adaptive regulation of the NRF2 defense system. Biomaterials. 2020;257:120264. doi: 10.1016/j.biomaterials.2020.120264. 32791387

[97] Zhao G, Liu F, Liu Z, et al. MSC-derived exosomes attenuate cell death through suppressing AIF nucleus translocation and enhance cutaneous wound healing. Stem Cell Res Ther. 2020;11(1):174. doi: 10.1186/s13287-020-01616-8. 32393338 PMC7212595

[98] Fan M, Li H, Shen D, et al. Decoy exosomes offer protection against chemotherapy-induced toxicity. Adv Sci (Weinh). 2022;9(32):e2203505. doi: 10.1002/advs.202203505. 36058003 PMC9661835

[99] Eshaq AM, Flanagan TW, Ba Abbad AA, et al. Immune checkpoint inhibitor-associated cutaneous adverse events: mechanisms of occurrence. Int J Mol Sci. 2024;26(1):88. doi: 10.3390/ijms26010088. 39795946 PMC11719825

[100] Allais BS, Fay CJ, Kim DY, et al. Cutaneous immune-related adverse events from immune checkpoint inhibitor therapy: Moving beyond “maculopapular rash. Immunol Rev. 2023;318(1):22–36. doi: 10.1111/imr.13257. 37583051

[101] Yi Y-F, Fan Z-Q, Liu C, et al. Immunomodulatory effects and clinical application of exosomes derived from mesenchymal stem cells. World J Stem Cells. 2025;17(3):103560. doi: 10.4252/wjsc.v17.i3.103560. 40160689 PMC11947897

[102] Engeroff P, Vogel M. The potential of exosomes in allergy immunotherapy. Vaccines (Basel). 2022;10(1) :133. doi: 10.3390/vaccines10010133. PMC8780385 35062793

[103] Lyu C, Sun H, Sun Z, et al. Roles of exosomes in immunotherapy for solid cancers. Cell Death Dis. 2024;15(2):106. doi: 10.1038/s41419-024-06494-z. 38302430 PMC10834551

[104] Abdelhakim M, Ogawa R. Emerging therapies in chronic wound healing: advances in stem cell therapy, growth factor modulation, mechanical strategies and adjuvant interventions. Dermatol Ther (Heidelb). 2025;15(12):3533–3545. doi: 10.1007/s13555-025-01564-2. 41075105 PMC12619873

[105] Marques da Silva M, Olsson DC, Teixeira BL, et al. Mesenchymal stromal cell secretome for therapeutic application in skin wound healing: a systematic review of preclinical studies. Cells Tissues Organs. 2023;212(6):567–582. doi: 10.1159/000526093. 35871510

[106] Yan K, Lv H, Wen J, et al. PreTP-stack: prediction of therapeutic peptides based on the stacked ensemble learing. IEEE/ACM Trans Comput Biol Bioinform. 2023;20(2):1337–1344. doi: 10.1109/TCBB.2022.3183018. 35700248

[107] Butala S, Castelo-Soccio L, Seshadri R, et al. Biologic versus small molecule therapy for treating moderate to severe atopic dermatitis: clinical considerations. J Allergy Clin Immunol Pract. 2023;11(5):1361–1373. doi: 10.1016/j.jaip.2023.03.011. 36948491 PMC10164714

[108] Trigo CM, Rodrigues JS, Camões SP, et al. Mesenchymal stem cell secretome for regenerative medicine: where do we stand? J Adv Res. 2025;70:103–124. doi: 10.1016/j.jare.2024.05.004. 38729561 PMC11976416

[109] Trzyna A, Banaś-Ząbczyk A. Adipose-derived stem cells secretome and its potential application in “stem cell-free therapy”. Biomolecules. 2021;11(6):878. doi: 10.3390/biom11060878. 34199330 PMC8231996

[110] Feng H, Gong S, Liu J, et al. Adipose-derived stem cell exosomes: mechanisms and therapeutic potentials in wound healing. Biomark Res. 2025;13(1):88. doi: 10.1186/s40364-025-00801-2. 40542446 PMC12181847

[111] Gupta K, Perkerson RB, Parsons TM, et al. Secretome from iPSC-derived MSCs exerts proangiogenic and immunosuppressive effects to alleviate radiation-induced vascular endothelial cell damage. Stem Cell Res Ther. 2024;15(1):230. doi: 10.1186/s13287-024-03847-5. 39075600 PMC11287895

[112] Pintea A, Manea A, Pintea C, et al. Peptides: emerging candidates for the prevention and treatment of skin senescence: a review. Biomolecules. 2025;15(1):88. doi: 10.3390/biom15010088. 39858482 PMC11762834

[113] Skibska A, Perlikowska R. Signal peptides - promising ingredients in cosmetics. Curr Protein Pept Sci. 2021;22(10):716–728. doi: 10.2174/1389203722666210812121129. 34382523

[114] Badilli U, Inal O. Current approaches in cosmeceuticals: peptides, biotics and marine biopolymers. Polymers (Basel). 2025;17(6):798. doi: 10.3390/polym17060798. 40292641 PMC11946782

[115] Ash M, Zibitt M, Shauly O, et al. The innovative and evolving landscape of topical exosome and peptide therapies: a systematic review of the available literature. Aesthet Surg J Open Forum. 2024;6:ojae017. doi: 10.1093/asjof/ojae017. 38633728 PMC11023079

[116] Curkovic NB, Bai K, Ye F, et al. Incidence of cutaneous immune-related adverse events and outcomes in immune checkpoint inhibitor-containing regimens: a systematic review and meta-analysis. Cancers (Basel). 2024;16(2):340. doi: 10.3390/cancers16020340. 38254829 PMC10814132

[117] Nevid M, Boguniewicz M. Current and emerging biologics for atopic dermatitis. Immunol Allergy Clin North Am. 2024;44(4):577–594. doi: 10.1016/j.iac.2024.08.001. 39389711

[118] Ma L, Chen X, Aziz MAA, et al. Dual biologic therapy for psoriasis in a patient with atopic dermatitis. Psoriasis (Auckl). 2025;15:159–161. doi: 10.2147/PTT.S516268. 40290565 PMC12034285

[119] Chen J, Li P, Zhang T, et al. Review on strategies and technologies for exosome isolation and purification. Front Bioeng Biotechnol. 2021;9:811971. doi: 10.3389/fbioe.2021.811971. 35071216 PMC8766409

[120] Ranbhor R. Advancing monoclonal antibody manufacturing: process optimization, cost reduction strategies, and emerging technologies. BTT. 2025;ume 19:177–187. doi: 10.2147/BTT.S515078. PMC11994081 40226587

[121] Russo F, Galluzzo M, Stingeni L, et al. Long-term drug survival and effectiveness of secukinumab in patients with moderate to severe chronic plaque psoriasis: 42-month results from the SUPREME 2.0 study. Clin Cosmet Investig Dermatol. 2023;16:3561–3574. doi: 10.2147/CCID.S416149. PMC10725693 38107670

[122] Haykal D, Wyles S, Garibyan L, et al. Exosomes in cosmetic dermatology: a review of benefits and challenges. J Drugs Dermatol. 2025;24(1):12–18. doi: 10.36849/JDD.8872. 39761139

[123] Palakurthi SS, Shah B, Kapre S, et al. A comprehensive review of challenges and advances in exosome-based drug delivery systems. Nanoscale Adv. 2024;6(23):5803–5826. doi: 10.1039/d4na00501e. 39484149 PMC11523810

[124] Lee YS. Regenerative skin remodeling through exosome-based therapy: a case study demonstrating 21-month sustained outcomes in pore size, erythema, and hyperpigmentation. Dermatol Ther (Heidelb). 2025;15(10):3055–3064. doi: 10.1007/s13555-025-01501-3. 40770125 PMC12454781

[125] Humbert C, Cordier C, Drut I, et al. GMP-compliant process for the manufacturing of an extracellular vesicles-enriched secretome product derived from cardiovascular progenitor cells suitable for a phase I clinical trial. J Extracell Vesicles. 2025;14(8):e70145. doi: 10.1002/jev2.70145. 40831309 PMC12365392

[126] Alsaidan OA. Current trends in exosomes as therapeutic drug delivery systems. Naunyn Schmiedebergs Arch Pharmacol. 2026;399(4):4755–4782. doi: 10.1007/s00210-025-04615-9. 41143953

[127] Carrara SC, Ulitzka M, Grzeschik J, et al. From cell line development to the formulated drug product: the art of manufacturing therapeutic monoclonal antibodies. Int J Pharm. 2021;594:120164. doi: 10.1016/j.ijpharm.2020.120164. 33309833

[128] Bai G, Truong TM, Pathak GN, et al. Clinical applications of exosomes in cosmetic dermatology. Skin Health Dis. 2024;4(6):e348. doi: 10.1002/ski2.348. 39624733 PMC11608875

[129] Nahm WJ, Nikas C, Goldust M, et al. Exosomes in dermatology: a comprehensive review of current applications, clinical evidence, and future directions. Int J Dermatol. 2025;64(11):1995–2010. doi: 10.1111/ijd.17903. 40533901

[130] Serrano DR, Juste F, Anaya BJ, et al. Exosome-based drug delivery: a next-generation platform for cancer, infection, neurological and immunological diseases, gene therapy and regenerative medicine. Pharmaceutics. 2025;17(10):1336. doi: 10.3390/pharmaceutics17101336. 41155971 PMC12567338

[131] Domaszewska-Szostek A, Krzyżanowska M, Polak A, et al. Effectiveness of extracellular vesicle application in skin aging treatment and regeneration: do we have enough evidence from clinical trials? Int J Mol Sci. 2025;26(5):2354. doi: 10.3390/ijms26052354. 40076975 PMC11899913

[132] Bin Dayel S, Hussein RS. Exosomes in dermatology: emerging roles in skin health and disease. Pharmaceutics. 2025;17(5):600. doi: 10.3390/pharmaceutics17050600. 40430891 PMC12114925

[133] Li L, Wang F, Zhu D, et al. Engineering exosomes and exosome-like nanovesicles for improving tissue targeting and retention. Fundam Res. 2025;5(2):851–867. doi: 10.1016/j.fmre.2024.03.025. 40242543 PMC11997600

[134] Mondal J, Pillarisetti S, Junnuthula V, et al. Hybrid exosomes, exosome-like nanovesicles and engineered exosomes for therapeutic applications. J Control Release. 2023;353:1127–1149. doi: 10.1016/j.jconrel.2022.12.027. 36528193

[135] Chen Y, Qi W, Wang Z, et al. Exosome source matters: a comprehensive review from the perspective of diverse cellular origins. Pharmaceutics. 2025;17(2):147. doi: 10.3390/pharmaceutics17020147. 40006514 PMC11858990

[136] Li Q, Li Y, Shao J, et al. Exploring regulatory frameworks for exosome therapy: insights and perspectives. Health Care Sci. 2025;4(4):299–309. doi: 10.1002/hcs2.70028. 40861511 PMC12371722

[137] Verma N, Arora S. Navigating the global regulatory landscape for exosome-based therapeutics: challenges, strategies, and future directions. Pharmaceutics. 2025;17(8):990. doi: 10.3390/pharmaceutics17080990. 40871013 PMC12389065

[138] Volarevic V, Markovic BS, Gazdic M, et al. Ethical and safety issues of stem cell-based therapy. Int J Med Sci. 2018;15(1):36–45. doi: 10.7150/ijms.21666. 29333086 PMC5765738

